# Effects of Wind Speed on Water Uptake, Phase State, and Viscosity of Sea Spray Aerosols

**DOI:** 10.1021/acsearthspacechem.5c00215

**Published:** 2025-10-21

**Authors:** Chamika K. Madawala, Mengnan Sun, Carolina Molina, Raymond J. Leibensperger, Chathuri P. Kaluarachchi, Lincoln Mehndiratta, Ke’La A. Kimble, Greg Sandstrom, Charbel Harb, Grant B. Deane, M. Dale Stokes, Christopher Lee, Jonathan. H. Slade, Kimberly A. Prather, Vicki H. Grassian, Alexei V. Tivanski

**Affiliations:** 1 Department of Chemistry, 4083 University of Iowa, 230 N, Madison St., Iowa City, Iowa 52242, United States; 2 Department of Chemistry and Biochemistry, 8784 University of California San Diego, La Jolla, California 92093, United States; 3 Scripps Institution of Oceanography, 8784 University of California San Diego,La Jolla, California 92093, United States; 4 6856 RTI International, Durham, North Carolina 27713, United States

**Keywords:** sea spray aerosol, atomic force microscopy, single particle, wind speed, phase state, viscosity, water uptake

## Abstract

This study investigates the effects of wind speed on physicochemical properties such as water uptake, phase state, and viscosity at varying relative humidity (RH) of individual nascent sea spray aerosols (SSAs). We examined SSA sized within 0.1−0.6 μm generated from a wind-wave channel at two wind speeds: 10 m/s representing a wind lull scenario over the ocean and 19 m/s corresponding to wind speeds encountered in stormy conditions. Atomic force microscopy (AFM) was utilized to study two predominant SSA morphologies: core−shell and rounded. AFM phase state measurements at 60% RH revealed that shells of core−shells at 19 m/s were largely liquid, while those at 10 m/s were mostly semisolid or liquid with similar proportions, where semisolid shells exhibited higher viscosities at lower wind speed. Rounded SSAs were predominantly liquid or semisolid at 60% RH, with similar semisolid viscosities for both wind speeds. Increased water uptake was observed for core−shells at 19 m/s, while rounded SSA had similar hygroscopicity between the two wind conditions. Collectively, we observed a variation in the physicochemical properties of SSA generated at two wind speeds, which can be attributed to the impact of elevated wind speed on disrupting the sea surface microlayer film structure and composition.

## Graphical abstract

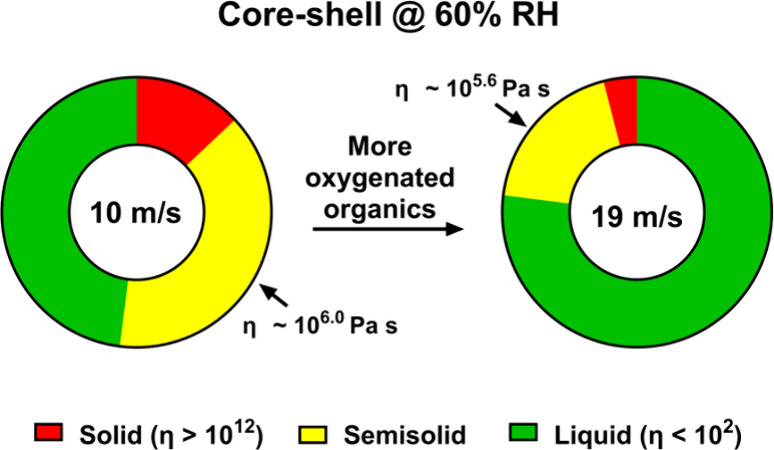

## Introduction

Exploring physicochemical properties such as water uptake, phase state, and viscosity of sea spray aerosols (SSAs) is important as they play a major role in regulating climate-relevant processes.^1–7^ SSAs impact radiative forcing by directly scattering solar radiation or indirectly affecting cloud properties by serving as cloud condensation nuclei (CCN) or ice nucleating particles (INPs).^8–12^ SSAs are produced when breaking waves entrain bubbles in seawater, which rise and burst at the air−water interface through wind-driven mechanisms.^13,14^ Organic, inorganic, and biological species dissolved in bulk seawater tend to partition at the uppermost sea surface microlayer (SML; approximately a submicrometer thick) and in turn selectively transfer into SSAs.^13,15–18^ Prior research has shown that the formation, thickness, and distribution of SML is significantly impacted by varying wind speed conditions.^16,19–21^ In particular, varying wind speeds can impact the film structure, distribution, and composition of SML, influencing SSA formation mechanisms, composition, and morphology.^7,16,19–23^ The chemical and morphological complexity of SSA in turn influence their radiative effects by governing key properties such as water uptake, phase state, and viscosity under different atmospheric relative humidity (RH) conditions.^24–27^

The effect of wind speed on the chemical composition of SSA has been studied previously.^7^ For example, it has been observed that as wind speed increases, the organic mass fraction in SSA decreases, while the formation of highly oxygenated compounds becomes more pronounced, especially for core−shell SSA.^7^ Thus, wind speed increase is expected to alter the chemical composition of SSA, consequentially influencing the extent of water uptake at a particular RH, which in turn modifies the solute concentration, ultimately impacting the phase state and viscosity of SSAs.^28,29^ In the context of phase state and viscosity, variable RH in the atmosphere results in a dynamic phase state (e.g., solid, semisolid, and liquid) and viscosity of SSAs, which in turn influences their interactions with atmospheric gases.^30^ In particular, solid or semisolid SSA may exhibit low reactivity with atmospheric gases due to the lowering of the diffusion coefficient.^31,32^ Furthermore, the aerosol viscosity affects the equilibrium time scale for the diffusion of atmospheric gas molecules into and out of aerosols. This, in turn, influences the rate and type of heterogeneous reactions (e.g., surface or bulk oxidation) and determines the aerosols’ efficiency to act as CCN or INPs.^31,33^ Therefore, it is important to accurately determine the phase state and viscosity of SSA as a function of RH. This is particularly significant for submicrometer SSA, as they have significantly longer lifetimes in the atmosphere than supermicrometer-sized aerosols.^34^ Currently, no studies have been performed to directly measure the phase state and viscosity of individual SSAs at subsaturated RH as a function of wind speed. Such single-particle measurements may be particularly important for real SSAs that often display significant particle-to-particle variability, as previously reported in regard to ice nucleation.^12^ The physicochemical properties of SSA generated from wave breaking of seawater were previously reported in several wave flume studies.^28,35,36^ However, to our knowledge, no previous studies have investigated the effects of wind speed on water uptake, phase state, and viscosity of SSA on a single-particle basis.

In this study, we identify the relationship between wind speed and various properties of individual SSA (size range of 0.1−0.6 μm), specifically SSA water uptake, phase state, and viscosity. A month-long mesocosm experiment, CHAOS (characterizing atmosphere ocean parameters in SOARS: the Scripps ocean-atmospheric research simulator), was carried out in summer 2022 where seawater was collected from the southern coast of California and different wind-wave interactions were simulated using a breaking wave analogue. Individual submicrometer nascent SSAs were substrate-deposited under various wind speed conditions for offline atomic force microscopy (AFM) characterization. These single-particle measurements (collected on the same day: August 15th) are compared between two distinct wind speed conditions, 10 m/s, representing a wind lull scenario, and 19 m/s, which is characteristic of wind speed over the Southern Ocean that is encountered during stormy conditions.^37–41^ The rounded and core−shell morphologies were compared in this study as they collectively account for the majority of SSA morphologies (63% for 10 m/s and 69% for 19 m/s) in both wind speed conditions.^7^ In addition, the remaining ∼30% of SSA exhibited prism-like, rod, rod-inclusion core−shell, and aggregate morphologies. While these less common morphologies were observed under both wind speed conditions, they were not the focus of the present analysis.^7^ To investigate the effect of wind speed on water uptake, phase state, and viscosity of individual SSA as a function of RH, AFM was employed. The results discussed herein focusing on the extent of water uptake, phase state, and viscosity of SSA as a function of wind speed for each of the two major morphological types were correlated with the chemical composition and AFM-infrared spectroscopy (AFM-IR) results reported previously for SSA collected on the same sampling day (August 15th) over the same size range.^7^ A significant variation in these physicochemical properties was determined, especially for core−shell SSA, underscoring the importance of incorporating such variability into future investigations toward a more accurate estimation of climate-related effects of sea spray aerosols.

## Materials and Methods

### SSA Generation and Subsequent Substrate Collection for Offline Single-Particle Studies

The filtered seawater from the Pacific Ocean floor at the end of the Scripps Institution of Oceanography (SIO) pier in La Jolla, CA was filled into a combined wind tunnel and wave channel during the summer of 2022. The wind speeds were measured at a height of 0.6 m above the water in SOARS and extrapolated to a 10 m height value using an approach described by Hsu et al.^42^ Throughout the manuscript, the wind speed values correspond to those extrapolated at 10 m height values. The SSAs were generated on August 15th under two different wind conditions of 10 and 19 m/s, both atop a single wave field. Additional details of the SSA generation and wind speed measurements including the dimensions and setup of the wind-wave channel as well as wind speed measurement techniques using an anemometer can be found elsewhere.^7,43^ A micro-orifice uniform deposit impactor (MOUDI; MSP, Inc., model 125R) at a flow rate of 10 L/min was used to deposit individual submicrometer SSA onto hydrophobically coated (Rain-X) silicon substrates (Ted Pella, Inc.) that were placed on several selected MOUDI stages with deposition at ca. 50% relative humidity (RH).^35^ MOUDI stages 7, 8, and 9 were used, corresponding to 50% cutoff aerodynamic diameter ranges of 0.32−0.56, 0.18−0.32, and 0.10−0.18 μm, respectively. The substrate-deposited SSA samples were stored in clean Petri dishes and kept inside a laminar flow hood (NuAire, Inc., NU-425−400) at an ambient temperature (20−25 °C) and pressure for 2−3 months prior to AFM experiments. No unexpected or unusually high safety hazards were encountered.

### Single-Particle AFM Imaging to Determine the Morphologies of SSA at 20% RH

A molecular force probe 3D AFM (Asylum Research, Santa Barbara, CA) was used for imaging individual substrate-deposited nSSA at ambient temperature (20−25 °C) as described in prior studies.^7,28,35,44,45^ A custom-made humidity cell was used to control RH with a range of 20−80%.^25^ Silicon nitride AFM tips (MikroMasch, model CSC37, typical tip radius of curvature of ∼10 nm, nominal spring constant of 1.0 N/m) were used for imaging and force spectroscopy measurements. Prior to the AFM imaging of substrate-deposited SSA, a hydration−dehydration cycle was carried out to ensure the proper restructure of previously deposited particles at 50% RH where the humidity was first increased to ∼80% RH, which resulted in deliquescence of the particles and then allowed at least 10 min of equilibrium time to ensure that SSAs are in thermodynamic equilibrium with surrounding water vapor. Then, the RH was slowly decreased to ∼20% RH, resulting in the dehydration of the SSAs for imaging the particles.^46^ The selection of these two RH values is based on the deliquescence and efflorescence RH for pure NaCl that occur at ∼75 and ∼40%, respectively.^46,47^ The AFM AC (intermittent contact) imaging mode was used to collect 3D height and phase images of individual SSA to determine their morphology and volume-equivalent diameter as described previously.^28,44,48^

### AFM Measurements of SSA Water Uptake at 80% RH and Phase States at 20 and 60% RH

The analysis of the 3D growth factor (GF) at 80% RH was employed to quantify the water uptake properties of SSA on a single-particle basis. The GF is defined as the ratio of the volume-equivalent diameter of an individual SSA at 80% RH over the corresponding volume-equivalent diameter recorded at 20% RH, where higher values would indicate the presence of more hygroscopic components.^28,49,50^ The GF measurements were performed on approximately eight individual SSAs with the two most abundant morphologies (core−shell and rounded) observed at wind speeds of 10 and 19 m/s, at the highest relative occurrence size ranges of 0.2−0.5 and 0.1−0.2 μm, respectively, and the values were reported as an average and one standard deviation.

AFM was employed to identify the phase state at 20 and 60% RH under ambient temperature (20−25 °C) and pressure for SSA with the most abundant morphologies (i.e., core−shell and rounded) using a previously reported method.^25,28,35,48^ The RH values were selected as a benchmark based on sucrose that shows solid-to-semisolid and semisolid-to-liquid phase transitions at ∼20 and 60% RH, respectively.^25,28,48^ A maximum force of 20 nN and scan rate of 1 Hz were used.^28,35^ At least five force plots were collected per individual SSA by probing at the shell region of the core−shell and at approximately the center of the rounded SSA.^28^ The collected force plots were then used to quantify the viscoelastic response distance (VRD, nm) and relative indentation depth (RID, the ratio of the indentation distance over the particle height) for an individual particle at 20 and 60% RH.^25,35^ The single-particle phase state identification was conducted using an established framework based on the VRD and RID measurements, as described in prior studies.^25,48^ The VRD values measured on SSA in the semisolid phase state were reported as an average and one standard deviation. Approximately 15 or more individual SSAs for each wind speed and for each morphology were investigated. The VRD values and relative abundance (i.e., an average and one standard deviation for fraction of particles) of phase states for the shell of core−shell SSA and rounded particles were recorded at a volume-equivalent diameter range of 0.1−0.6 μm at two wind speeds of 10 and 19 m/s.

As the total number of individual particles that can be reasonably studied with AFM is somewhat limited, we utilized a statistical probability distribution analysis to assess the statistical significance of the AFM-based phase state measurements.^28^ The detailed description of the approach can be found elsewhere.^51,52^ Briefly, the probability distributions associated with the likelihood of sampling one of the three phase states were generated using a self-coded Monte Carlo-like simulation method for a “true” population of 10,000 particles.^51–53^ The average with one standard deviation for the fraction of particles from each phase state was obtained by fitting the probability distribution plots with the Gaussian function.^28^ The results were recorded for both wind conditions at 10 and 19 m/s as a function of RH.

### AFM Measurements of Semisolid SSA Viscosity at RH 60%

Viscosity quantification at 60% RH was performed using AFM under ambient temperature (20−25 °C) and pressure for core−shell and rounded morphologies. It should be noted that the methodology is specifically applicable for the quantification of viscosity in semisolid individual SSA. Thus, 60% RH was selected as a benchmark since previous phase state studies on sucrose showed the semisolid-to-liquid phase transition at 60% RH, which corresponds to a viscosity of 10^2^ Pa s.^45^ At least five force profiles were collected for each SSA by probing at the shell region of each core−shell and at an approximate center of rounded SSA. A previously reported method was then utilized to quantify the viscosity of each particle at 60% RH.^45^ At least five individual semisolid SSAs for each morphology type (core−shell and rounded) and for each wind speed were investigated for the viscosity quantification at the highest relative occurrence size ranges of 0.2−0.5 and 0.1−0.2 μm, respectively, under both wind speed conditions of 10 and 19 m/s.

## Results and Discussion

Figure 1A and Figure 1B show representative AFM 3D height images of the two main morphological SSA categories: rounded and core−shell, respectively, identified for SSA at 20% RH, for both 10 and 19 m/s wind speed conditions within a volume-equivalent diameter range of 0.1−0.6 μm. The qualitative analysis using AFM 3D height and phase images was used for the classification of SSA morphologies as described previously.^28,35,48^ The combined fraction of core−shell and rounded SSA accounts for 70% for both 10 and 19 m/s wind speed conditions. Thus, all results and corresponding discussion presented below and related to the impact of varying wind speed conditions on the water uptake, phase state, and viscosity will focus on these two predominant morphologies generated at two wind speeds.

**Figure 1:**
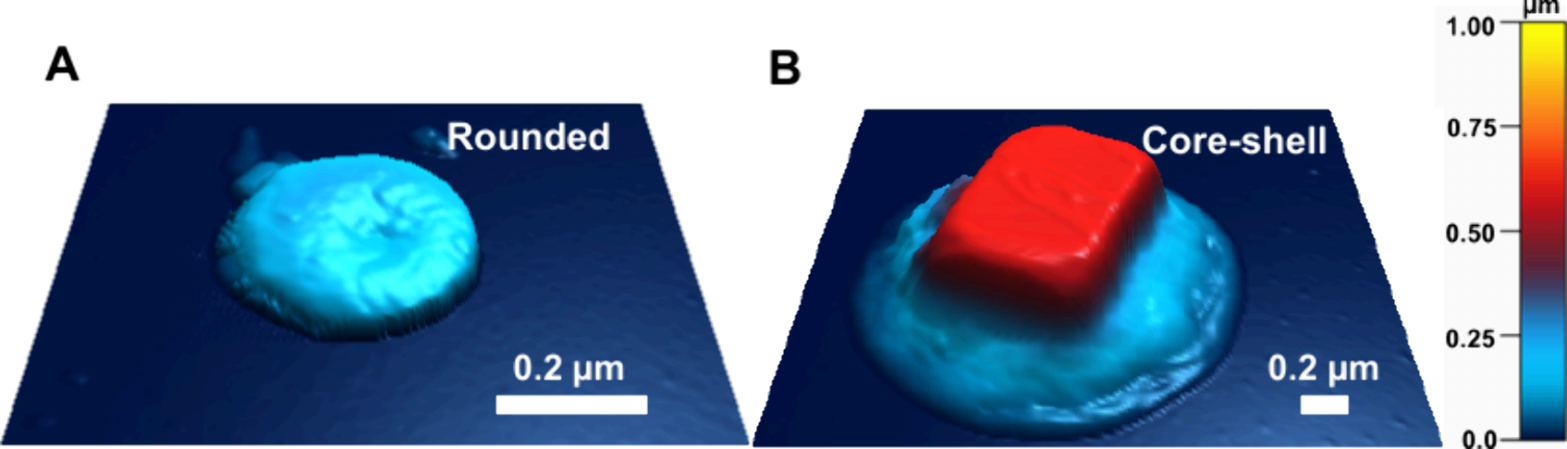
Representative AFM 3D height images at 20% RH of the two main morphological categories: (A) rounded and (B) core−shell SSA.

### Impact of Wind Speed on Water Uptake of Core−Shell and Rounded SSA

The measured 3D growth factor (GF) and corresponding hygroscopic parameter (κ_mixture_) of core−shell and rounded SSA at 10 and 19 m/s wind speeds were determined at 80% RH using a previously reported approach, and the corresponding average and one standard deviation values are reported in Table 1.^28,35,50,54,55^ The 3D growth factor was quantified by taking the ratio of volume-equivalent diameter of SSA from AFM imaging at the corresponding RH over that at dry RH. The hygroscopicity parameter (κ) was then calculated using the κ-Köhler framework, which relates the measured growth factor at a given RH to the equilibrium water activity. The 80% RH was selected because it is above the deliquescence point of pure NaCl (∼75% RH), thus assuming that the core of core−shell SSA is primarily NaCl, and such particles are expected to undergo a complete deliquescence at 80% RH to form liquid droplets.^50^ The measurements were performed on core−shell and rounded SSA at size ranges of 0.2−0.5 and 0.1−0.2 μm, respectively, considering the highest relative occurrence size range for each morphology as determined and reported previously.^7^ Specifically, the GF (range of 1.5−1.8) and κ_mixture_ (average 0.8 ± 0.2) values for core−shell SSA at 19 m/s wind speed were higher compared to the GF (range of 1.1−1.5) and κ_mixture_ (average 0.3 ± 0.2) values of core−shells at 10 m/s wind speed. The range of GF and κ_mixture_ values determined at 80% RH in this study for core−shell SSA at 19 m/s aligns well with SSA-relevant NaCl, which exhibits a GF and κ_mixture_ of 1.8 and 1.2, respectively.^56^ The lower water uptake observed for core−shell SSA relative to pure NaCl is expected due to the presence of less hygroscopic organic shells. A significant increase in hygroscopicity (higher GF) observed on core−shell SSA at 19 m/s wind speed is consistent with the AFM-IR spectral data and AFM phase state measurements reported previously,^7^ which showed the presence of more oxygenated organics and increasing relative abundance of liquid shells at elevated wind speed. In contrast, water uptake measurements on rounded SSA at two wind speeds showed no apparent differences, where GF (range of 1.0−1.3) and κ_mixture_ (average 0.1 ± 0.1) values at 10 m/s were similar to GF (range of 1.0−1.3) and κ_mixture_ (average 0.1 ± 0.1) values for SSA generated at 19 m/s wind speed. The results are consistent with AFM-IR spectral data reported previously, which had similar functional groups for rounded SSA generated at both wind speeds.^7^ The observed GF and κ_mixture_ values were consistent with previous studies on model and nascent SSA, encompassing both core−shell and rounded SSA morphologies.^35,50,54,57^ In particular, the range of GF and κ_mixture_ values determined in this study overlaps well with those observed for pure organic systems such as sucrose, glucose, and malonic acid,^25,50^ as well as SSA-relevant two-component systems of NaCl/sodium alginate and NaCl/liposaccharides at various mass fractions.^54,58^

**Table 1:** Summary of Wind Speed Designation for Core−Shell and Rounded SSA Particles at 20 and 60% RH and Relative Distributions of Solid, Semisolid, and Liquid Phase States for the Shell of Core−Shell and Rounded SSA within a Volume-Equivalent Diameter Range of 0.1−0.6 μm^b^

		fraction of SSA at a particular phase state (%)							
wind speed	RH (%)	solid	semisolid	liquid	average GF at 80% RH	GF range at 80% RH	average **κ**_mixture_ at 80% RH	average viscosity at 60% RH, Pa s^a^	viscosity range at 60% RH, Pa s^a^
core−shell									
10 m/s	20	78 ± 11	16 ± 9	6 ± 1					
	60	13 ± 10	39 ± 12	48 ± 14	1.3 ± 0.1	1.1−1.5	0.3 ± 0.2	10^6.0 ± 0.3^	10^6.4^ to 10^5.6^
19 m/s	20	65 ± 14	23 ± 12	12 ± 10					
	60	4 ± 1	19 ± 12	77 ± 13	1.6 ± 0.1	1.5−1.8	0.8 ± 0.2	10^5.6 ± 0.6^	10^6.2^ to 10^5.1^
rounded									
10 m/s	20	25 ± 15	75 ± 17	0					
	60	0	73 ± 18	27 ± 17	1.1 ± 0.1	1.0−1.3	0.1 ± 0.1	10^6.1 ± 0.4^	10^6.6^ to 10^5.8^
19 m/s	20	36 ± 19	64 ± 19	0					
	60	0	82 ± 18	18 ± 16	1.1 ± 0.1	1.0−1.3	0.1 ± 0.1	10^6.4 ± 0.3^	10^6.6^ to 10^5.9^

a Viscosity data were determined for semisolid SSA only.

b The measurements of volume-equivalent growth factor (GF), hygroscopicity parameter (κ_mixture_), and viscosity range (η) at 60% RH were performed on the core−shell and rounded SSA at the highest relative occurrence size ranges of 0.2−0.5 and 0.1−0.2 μm, respectively.

### Impact of Wind Speed on the Phase State at 20 and 60% RH and Viscosity of Core−Shell SSA at 60% RH

Phase state identification on the two highest abundance morphologies of SSA was performed at 20 and 60% RH using AFM (i.e., force profiles).^25,28,35,48^ The measurements over the core of core−shell SSA particles were not reported because it is solid with possibly a thin organic layer, as shown in prior studies, and will not undergo a phase transition prior to reaching the typical deliquescence point of ∼75% RH for pure NaCl.^28,54^ The force profiles were then used to quantify VRD (nm, viscoelastic response distance) and RID (ratio of the indentation depth over the particle height) for an individual particle at a particular RH and determine phase states using previously established frameworks based on these measurements.^25,28,35,48^ As no apparent size-dependent phase state was observed for core−shells and rounded SSA, the phase state results for each particle type were combined over a wider volume-equivalent diameter range of 0.1−0.6 μm.

Figure 2A,B and Table 1 show the relative distributions of solid, semisolid, and liquid phase states for the shell region of core−shell SSA at 10 and 19 m/s. At 20% RH, shells of core−shell SSA exhibited all three phase states of solid, semisolid, and liquid shells, where most shells were solid under both wind conditions. However, shells of core−shells at 19 m/s showed a higher proportion of semisolid and liquid shells compared to those generated at 10 m/s wind speed at 20% RH. Furthermore, the VRD values measured on semisolid shells at 19 m/s (VRD: 0.5−3.6 nm) were greater than those for shells at 10 m/s (VRD: 0.5−3.1 nm), which is likely indicative of lower shell viscosity as a result of the increase in wind speed. As RH increased to 60%, shells of core−shell SSA at 19 m/s became hydrated and a significant fraction of shells were liquid, while shells of core−shells at 10 m/s continued with approximately similar fractions of semisolid and liquid shells with some shells retaining a solid phase state. Additionally, the VRD values measured on semisolid shells at 19 m/s (VRD: 1.3−11.8 nm) wind speed were greater than those under 10 m/s wind speed conditions (VRD: 0.5−6.5 nm).

**Figure 2:**
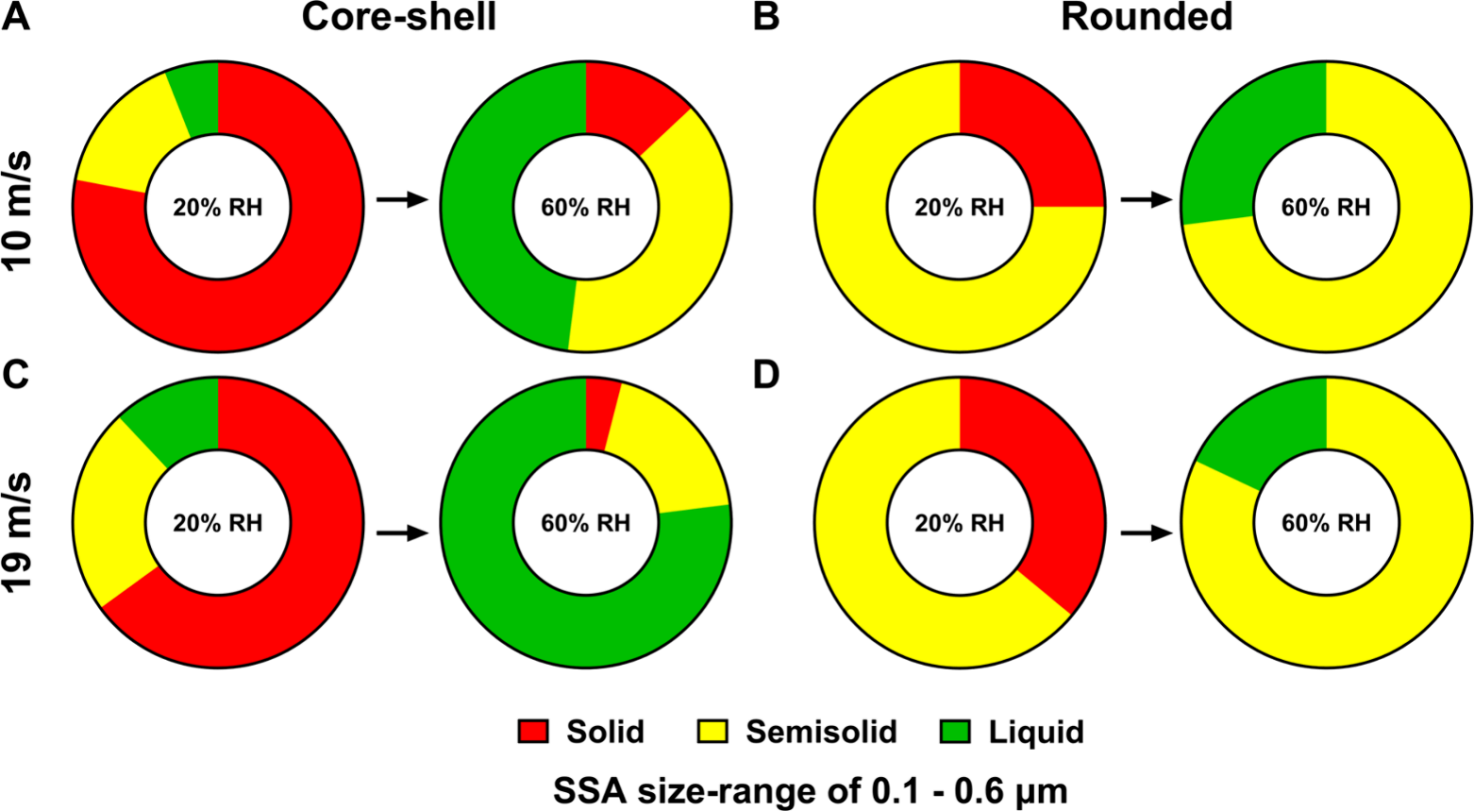
Relative distributions of solid, semisolid, and liquid phase states at 20 and 60% RH for shell regions of core−shells at 10 (A) and 19 m/s (C) wind speeds and rounded SSA at 10 (B) and 19 m/s (D) wind speeds. For both morphological types, the same SSA volume-equivalent diameter range of 0.1−0.6 μm was compared. Arrows are for illustrative purposes only.

The force profiles obtained over an individual particle were then utilized to simultaneously measure force as a function of indentation distance, and this data can be fitted to the AFM-based Kelvin−Voigt viscoelastic model to yield the particle viscosity using previously established frameworks based on these measurements.^45^ Table 1 shows the viscosity values measured on semisolid core−shell particles at 60% RH within the volume-equivalent diameter range of 0.2−0.5 μm. Figure 3A shows representative force versus indentation distance plots (symbols are data) as the tip approaches the particle surface measured at 60% RH over the shell region of core−shells (Figure 1B), along with the solid fit lines (red and black fit lines for 10 and 19 m/s, respectively) to yield viscosity values at 10 and 19 m/s, respectively. Specifically, the representative force plots (Figure 3A) collected at 60% RH on the shell region of core−shells with volume-equivalent diameters (*D*_vol_) of ∼300 nm at 10 m/s and ∼465 nm at 19 m/s yielded viscosities of 10^6.21 ± 0.04^ and 10^5.3 ± 0.1^ Pa s for 10 and 19 m/s wind speed conditions, respectively. Overall, it appears that the viscosity range for the shells of core−shells at 10 m/s was somewhat higher (ranging between 10^6.4^ and 10^5.6^ Pa s), compared to that of shells at 19 m/s, showing lower viscosities (ranging between 10^6.2^ and 10^5.1^ Pa s), which is consistent with the VRD values observed for shells of core−shells at each wind speed, indicative of lower shell viscosity with the increase in wind speed. The results are consistent with the presence of more oxygenated organic compounds, as evident by the AFM-PTIR measurements discussed previously.^7^ Collectively, due to the increase in wind speed, the viscosity of semisolid shells at 60% RH appears to decrease.

**Figure 3:**
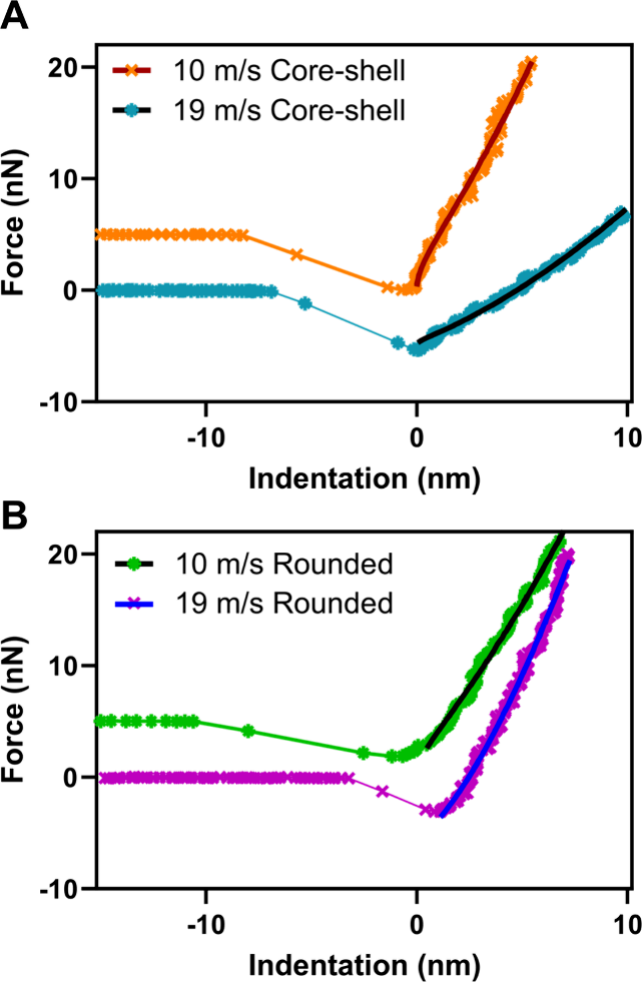
Representative force versus indentation distance (symbols) at 60% RH and corresponding fit (solid lines) for viscosity quantification using the AFM viscoelastic model^44^ for (A) core−shell SSA (orange crosses and red line at 10 m/s and blue asterisks and black line at 19 m/s) and (B) rounded SSA (green asterisks and black line at 10 m/s and purple crosses and blue line at 19 m/s). Only the approach to the particle surface data is shown. The force data at 10 m/s wind speed for each morphology type was offset by 5 nN for clarity.

### Impact of Wind Speed on the Phase State at 20 and 60% RH and Viscosity of Rounded SSA at 60% RH

Figure 2C,D and Table 1 show the relative distribution of solid, semisolid, and liquid phase states for rounded SSA under 10 and 19 m/s wind speed conditions. Specifically, at 20% RH, rounded SSAs under both wind conditions were either solid or semisolid where majority of rounded SSAs were semisolid in the phase state. Additionally, the VRD values measured on semisolid rounded SSA at 60% RH at 10 and 19 m/s were similar (VRD range of 0.9−6.3 nm at 10 m/s and 0.6−6.1 nm at 19 m/s), which is consistent with the presence of similar functional groups for each sample, as evident by the AFM-PTIR measurements discussed previously.^7^ As RH increased to 60%, rounded SSAs under both wind conditions were either semisolid or liquid, while the majority of rounded SSAs were semisolid with a small fraction as a liquid. Figure 3B shows representative force versus indentation distance plots (symbols are data) as the tip approaches the particle surface measured at 60% RH over an approximate center of rounded particles along with the solid fit lines (black and blue fit lines for 10 and 19 m/s, respectively) to yield viscosity values at 10 and 19 m/s, respectively. Specifically, the representative force plot (Figure 3B) collected on the rounded SSA at 60% RH yielded viscosities of 10^6.04 ± 0.04^ Pa s (*D*_vol_ ∼165 nm) and 10^6.2 ± 0.2^ Pa s (*D*_vol_ ∼200 nm) for 10 and 19 m/s wind speed conditions, respectively. Overall, the viscosity for the rounded SSA was comparable between the two wind speeds of 10 and 19 m/s, showing a viscosity range of 10^6.6^ to 10^5.8^ Pa s for both wind conditions, which is consistent with the AFM-PTIR measurements for rounded SSA at each wind speed, confirming the presence of similar functional groups under both wind conditions of 10 and 19 m/s.^7^

## Summary and Implications

The water uptake, phase state, and viscosity of individual SSA collected under two wind speed conditions were directly measured by using atomic force microscopy at various relative humidity values. Among different morphologies identified, approximately 70% of SSA was core−shell and rounded under both wind conditions of 10 and 19 m/s, and thus these two morphologies are the focus of this current study. The results show significant variability in these physicochemical properties with respect to the RH and wind speed conditions. As demonstrated in the current study, increased hygroscopicity was observed for SSA core−shells at 19 m/s, which had more oxygenated organic species, while rounded SSA had similar hygroscopicity during both wind conditions, consistent with a similar composition. Furthermore, higher hygroscopicity and more efficient water uptake properties were observed for core−shell SSA compared to rounded SSA. Varying hygroscopicity would in turn impact the size of SSA at a particular atmospheric RH and thus modify their light scattering ability (e.g., Mie scattering).

The AFM phase state measurements at 20% RH revealed that an increase in wind speed from 10 to 19 m/s resulted in an increase in the relative abundance of semisolid and liquid shells for core−shell SSA, while rounded SSA had approximately similar relative abundance of solid and semisolid phases. As RH increased to 60%, shells of core−shell and rounded SSA uptake water, becoming less viscous, and most of their corresponding phase states change into semisolid or liquid. No apparent differences were observed in the phase state and viscosity of rounded SSA between wind speeds. The shells of core−shell SSA appeared to have somewhat lower viscosities at 19 m/s at 60% RH, likely caused by higher hygroscopicity due to the presence of more oxygenated organic compounds when compared with the predominant aliphatic organic composition of shells of core−shell SSA at a 10 m/s wind speed. The observed variation in the viscosity and phase state of SSA at 10 and 19 m/s could be predominantly attributed to the change in the composition of SSA as shown in our previous study,^7^ which can be influenced by the changes in the structure and composition of SML at varying wind speeds. The phase state and viscosity findings align well with the previously reported AFM-PTIR data, indicating the presence of organics with similar compositions, including aliphatic and oxygenated species for rounded SSA at both wind speeds, consistent with no apparent variability in the phase state and viscosity at 10 and 19 m/s. In contrast, the shells of core−shell SSA displayed a wind speed-dependent composition where predominantly oxygenated organics were present under higher wind speed conditions, which is consistent with observed changes in their properties.^7^

The wind speed effects on the phase state and viscosity of SSA can facilitate a better understanding of their climate-relevant effects such as CCN ability and light scattering efficiency. As our results demonstrate significant variability of the SSA phase state and viscosity specifically for shells of core−shell SSA with respect to different wind conditions, the time scale to undergo chemical aging will be different. Specifically, for the core−shells analyzed between the two wind conditions of 10 and 19 m/s, the change in viscosity from 10^6.1^ to 10^5.6^ (the average viscosity for an average particle size of 300 nm) is expected to change the diffusion time scale of water molecules within core−shell SSA from 8 to 2 months (note, the corresponding diffusion time scales will be significantly lower for smaller SSA).^30^ Thus, this influences the rate and in some cases types of heterogeneous reactions (e.g., surface vs bulk oxidation) and extent of atmospheric aging and subsequently their ability to act as efficient CCN or INPs.^31,33^ In addition, the phase states may affect the diffusion length of different atmospheric gases due to the change in the viscosity coefficient of SSA at a particular atmospheric RH. For example, during atmospheric chemical aging, the characteristic mass-transport time of different atmospheric gases strongly depends on a particular phase state and viscosity of SSA.^31,59^ Therefore, a change in the reactive uptake probabilities may also alter the hygroscopic properties of SSAs, which can change the strength of the direct radiative forcing.^28,58^

Overall, we previously demonstrated the likely impact of varying wind speeds on the SML film structure and composition that in turn influences the SSA generating mechanisms and subsequently causes the variability in the morphology and composition of SSA.^7^ Specifically, at 10 m/s, the SML structure is intact and enriched with aliphatic compounds.^60^ However, the increase in wind speed to 19 m/s, wave breaking, and increased turbulence causes the disruption of the SML structure, leading to a more homogeneous water column in which the interfacial molecules are contained in the SML mix with the underlying more water-soluble compounds.^7^ Building on these findings, our current results clearly illustrated that this variability in SSA morphology and composition, which could be due to SML disruption at elevated wind speeds, directly impacts their physicochemical properties. In particular, our results indicate that higher wind speeds can cause significant changes in water uptake, viscosity, and phase state of SSA, emphasizing the importance of considering the effect of wind speed in accurate quantification and prediction of climate-relevant effects.
